# An fMRI Dataset for Concept Representation with Semantic Feature Annotations

**DOI:** 10.1038/s41597-022-01840-2

**Published:** 2022-11-24

**Authors:** Shaonan Wang, Yunhao Zhang, Xiaohan Zhang, Jingyuan Sun, Nan Lin, Jiajun Zhang, Chengqing Zong

**Affiliations:** 1 National Laboratory of Pattern Recognition, Institute of Automation, CAS, Beijing, China; 2 School of Artificial Intelligence, University of Chinese Academy of Sciences, Beijing, China; 3 CAS Key Laboratory of Behavioural Sciences, Institute of Psychology, Beijing, China; 4 Department of Psychology, University of Chinese Academy of Sciences, Beijing, China

**Keywords:** Neural encoding, Language

## Abstract

The neural representation of concepts is a focus of many cognitive neuroscience studies. Prior works studying concept representation with neural imaging data have been largely limited to concrete concepts. The use of relatively small and constrained sets of stimuli leaves open the question of whether the findings can generalize other concepts. We share an fMRI dataset in which 11 participants thought of 672 individual concepts, including both concrete and abstract concepts. The concepts were probed using words paired with images in which the words were selected to cover a wide range of semantic categories. Furthermore, according to the componential theories of concept representation, we collected the 54 semantic features of the 672 concepts comprising sensory, motor, spatial, temporal, affective, social, and cognitive experiences by crowdsourcing annotations. The quality assessment results verify this as a high-quality neuroimaging dataset. Such a dataset is well suited to study how the brain represents different semantic features and concepts, creating the essential condition to investigate the neural representation of individual concepts.

## Abstract (summary)

**Table:** 

Measurement(s)	Blood Oxygen Level-Dependent Functional MRI
Technology Type(s)	Functional Magnetic Resonance Imaging
Factor Type(s)	none
Sample Characteristic - Organism	Homo sapiens

## Background & Summary

Concepts are the most fundamental unit of human cognition, which abstracts and generalizes the common essential characteristics of perceived things from perceptual knowledge to rational knowledge. Humans formulate knowledge of the outside world and communicate their thoughts with others using symbolic language based on the learning and representation of concepts. The processing and storage of concepts are thought to be performed in the brain semantic system. However, it is not clear how exactly the semantic system represents concepts.

Previous neuroimaging research has gradually converged on several brain areas associated with different aspects of concept processing and representation. For instance, the inferior parietal lobe and much of the temporal lobe have been found to be involved in multiple perceptual processing, such as motion, sound, color, olfaction, and gustatory processing^1,2^. Neurons in the human medial temporal lobe (MTL) have been implicated in the representation of animals, objects or scenes^3^. However, to ensure the quality of scanned brain images, multiple repetitions and sufficient separations for each stimulus must be ensured, which limits the number of different stimuli collected in one experiment. Consequently, these previous studies only used a relatively small number of stimuli that are constrained in semantic space.

To comprehensively explore concept representation, we describe and share a functional magnetic resonance imaging (fMRI) dataset called CRSF (concept representation with semantic features)^4^ in which participants were asked to think of 672 individual concepts that cover a large semantic space. We select concepts from the Synonymy Thesaurus published by the Harbin Institute of Technology (HITST). There are 77,343 Chinese words in HITST, covering a major part of modern Chinese vocabulary. With a specifically defined selection procedure, we selected words from HITST that reflect its broad semantic categories. Each word is further paired with 6 related images to guide the participants’ attention to think about the concept. The diversity and scale of the presented dataset enable future fine-grained analysis of the brain representations of a broad range of concepts, categories, and semantics.

Moreover, we collected 54 semantic features of 672 words comprising sensory, motor, spatial, temporal, affective, social, and cognitive experiences. Each semantic feature of 672 words is evaluated with ratings on a 1–7 scale by a crowdsourced survey and annotated by 30 unique participants. These semantic features are summarized by Binder *et al*. (2016) based on previous neurobiological findings and are verified to capture important distinctions between a priori ontological types and categories, following the method proposed by Binder *et al*.^5^. Note that we exclude the 13 features of the original 67 semantic features proposed by Binder *et al*.^5^, i.e., motion, biomotion, shape, texture, audition, low, high, speech, time, social, harm, pleasant, and unpleasant because they have a very high correlation (Pearson correlation >0.8) with at least one of the other features.

In summary, this neuroimaging dataset with semantic annotations covers various concepts and semantic features. Therefore, it can be used to study a variety of research questions involving conceptual representations and is highly flexible with many different analyses^6,7^. We welcome laboratories and researchers from different backgrounds to explore this dataset in their own community and address specific questions.

## Methods

### Participants

For the MRI data collection, 18 participants (8 females, mean age 23.83 years ± 2.4 SD) were recruited, and the data of 7 participants were excluded because they did not complete all visits (mean 1.43 visits ± 0.73 SD). For the semantic feature annotation, 126 participants were recruited (72 females, mean age 22.72 years ± 2.13 SD), and each participant could complete as many surveys as they wanted to (mean 12.86 ± 8.53 SD) as long as they passed the quality evaluation (see Experimental Procedures for details) every time. Those who failed the quality evaluation once (24 participants) were not allowed to complete more surveys, and the failed surveys were not included in the analysis. All participants were native Chinese speakers, had normal or corrected-to-normal vision and were paid. They all provided written informed consent to the study and to the release of their data. The study was approved by the Institute of Psychology of the Chinese Academy of Sciences.

### Stimuli

One key challenge of stimulus selection is the coverage of its semantic space. We select words from the Synonymy Thesaurus published by the HITST at https://www.ltp-cloud.com/download#down_cilin. There are 77,343 Chinese words in HITST, covering a major part of modern Chinese vocabulary. The format and structure of HITST are similar to WordNet^8^. The words in HITST are organized in a tree structure. Synonyms and related words are collected under the same entry. One entry is labeled with an 8-digit code, denoting a 5-layer semantic category. The granularity of the semantic category becomes finer from the higher digit to the lower. To ensure the coverage of the selected words, we begin with the median digit, which is the third level of HITST’s semantic category. All entries with the same four highest digits were collected, and then the occurrence frequency of all words in the entries was calculated. We selected the one most frequent word in each entry and obtained 672 words. Since we selected words from all semantic categories of HITST with no bias, the chosen words should cover a broad semantic space.

In addition, each word was paired with 6 different images in the experiment. To obtain corresponding images that represent the meaning of the word, we used Baidu Search to query the target word and choose images manually.

### Experimental procedures

**Fig. 1:**
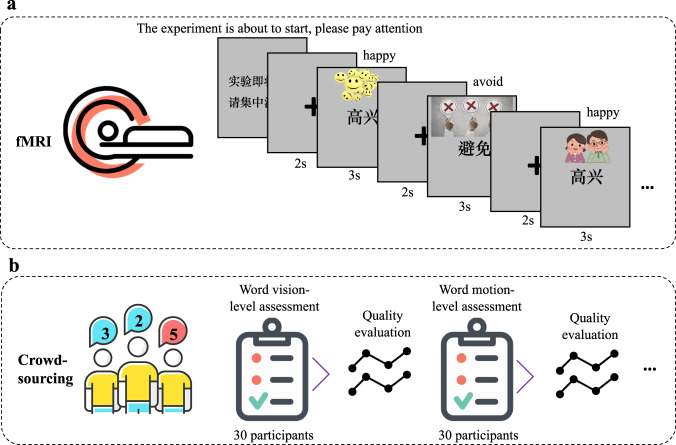
Schematic overview of the study procedure. (**a**) The participants followed the instructions on the screen and read the word stimuli while their brain activity was recorded by fMRI. (**b**) Participants rate semantic features of a word by questionnaires, and then quality evaluation is conducted to eliminate unqualified participants.

Before each scan, participants first completed a simple informational survey form and an informed consent form. During fMRI scanning, the participants were instructed to read attentively the presented words and think about their related concepts with the guidance of the accompanying images. Stimulus presentation was implemented using Psychtoolbox-3. Specifically, as shown in Fig. 1a, at the beginning of each run, the instruction “The experiment is about to start, please pay attention” appeared on the screen, followed by a fixation screen for 2 seconds. Then, each stimulus was presented for 3 s followed by a 2 s fixation period. The fMRI recording was split into 4 visits for sub01-sub05 and 6 visits for sub06-sub11. Within each scanning session, the 672 words were divided into four or six sets of 168 or 112 words. (done separately for each participant) and distributed across 12 runs. Each participant saw 6 repetitions of a word with a different picture, and it took 2 runs to see a single repetition of the whole 168 or 112 words (i.e., 84 or 56 words in a single run). Each run thus took 450 s for sub01-sub05 and 310 s for sub06-sub11. Please see the presentation scripts and onset files at https://openneuro.org/datasets/ds004301^4^ for more details.

For semantic feature annotation, we used the questionnaire collection platform at https://www.wjx.cn/ and recruited college students to participate. The participants were given the 672 words and instructions such as: “To what degree do you think of this thing as a characteristic or defining visual texture or surface pattern? (for the attribute *Pattern*)” with some examples. To avoid invalid surveys and ensure the effectiveness of the data, for each feature, we collected 30 surveys (with each survey include all 672 words) and calculated the correlation between the ratings of each participant and the mean ratings of the remaining participants using the reliability analysis tool in Jamovi (https://www.jamovi.org/). If the correlation was lower than 0.5, then we excluded the data of this participant and supplemented the results of a new participant until each feature has 30 valid surveys.

### Data acquisition

All functional and structural volumes were acquired using a 3T GE Discovery MR750 scanner at the Magnetic Resonance Imaging Research Center of the Institute of Psychology of the Chinese Academy of Sciences (IPCAS). Functional blood oxygenation level-dependent (BOLD) data were collected with gradient-echo echo-planar imaging in an interleaved fashion in 42 near-axial slices with a resolution of 3.0-mm isotropic voxels: TR = 2000 ms; TE = 30 ms, flip angle = 70, matrix size = 64 × 64, slice thickness = 3 mm and slice spacing = 0 mm. After half of the runs were performed during the first fMRI visit, T1 weighted structural volumes were collected with a single run lasting 5 minutes. T1-weighted structural images are obtained with a spoiled gradient-recalled pulse sequence in 176 sagittal slices with 1.0-mm isotropic voxels.

### MRI Preprocessing

MRI data, including anatomical and functional images, were automatically preprocessed using fMRIPrep^9^ (version 20.2.1, RRID:SCR_016216). As an automated and highly integrated tool, fMRIPrep has the ability to utilize the most suitable preprocessing workflow by autonomously regulating the properties of the dataset (seized by the metadata), which is regarded as a powerful fMRI reprocessing pipeline that enlists tools from distinguished neuroimaging software.

Specifically, before running fMRIPrep, we first transformed the raw DICOM files images to NIFTI files using dcm2niix at https://github.com/rordenlab/dcm2niix and then modified them to the Brain Imaging Data Structure (BIDS). While running fMRIPrep, the T1w-weighted data were skull-stripped; brain tissue was segmented into gray matter (GM), white matter (WM) and cerebrospinal fluid (CSF) on the brain-extracted T1w-weighted, brain surfaces were reconstructed using FreeSurfer, and volume-based spatial normalization to the standard space was performed through nonlinear registration. For functional MRI data, the following preprocessing was performed on each run per participant separately. First, a customized procedure of fMRIPrep with default settings was employed to generate a reference volume and its skull-stripped version. Then, FreeSurfer was employed to coregister the BOLD reference to the T1w-weighted reference. Last, BOLD runs were corrected for slice-time and were resampled to a volume-based standard space.

Based on the preprocessed BOLD data, various confounding time series were computed, which included DVARS, framewise displacement (FD), and three regionwise global signals. For each functional run, DVARS and FD were computed utilizing Nipype^10^. See the html reports at https://openneuro.org/datasets/ds004301^4^ generated by fMRIPrep for more details.

### Annotations

**Fig. 2:**
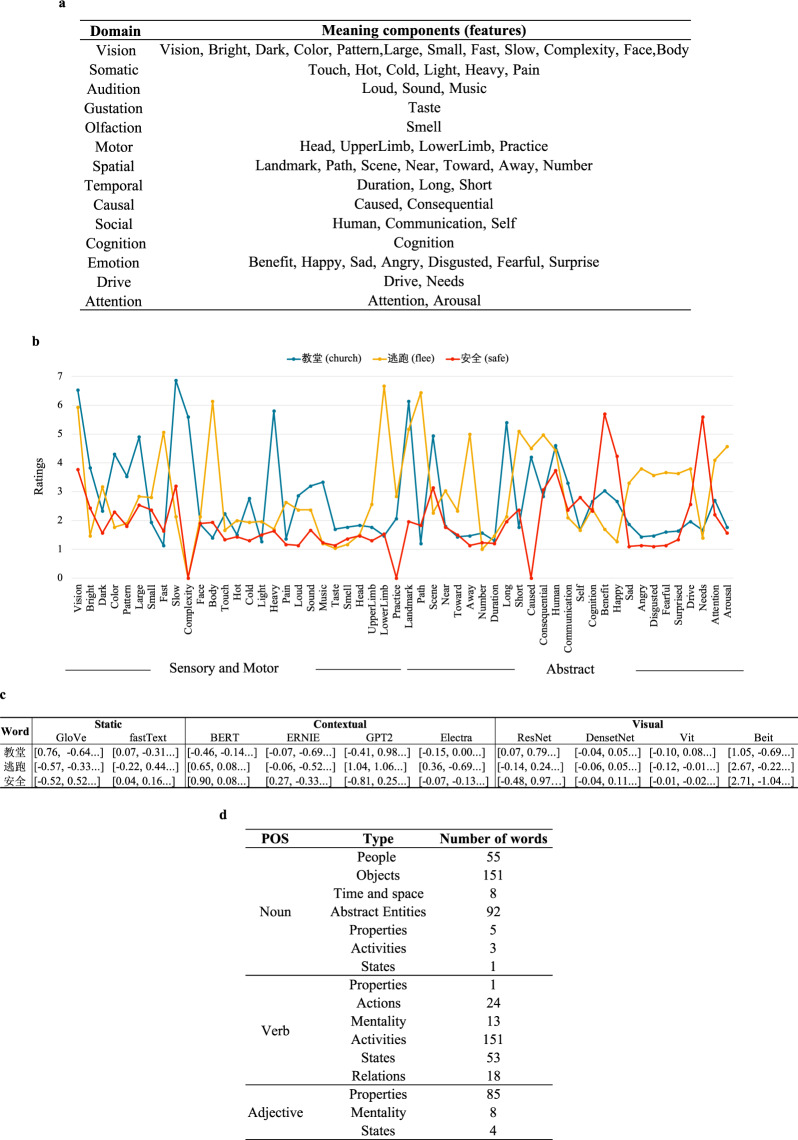
An example of annotation information for the stimuli. (**a**) Domains and meaning components in the semantic feature dataset. (**b**) Semantic feature ratings. (**c**) Word embeddings. (**d**) Part-of-speech tags and categories.
- **Static word embeddings** We used the fastText model^11^ and GloVe model^12^ to compute static word embeddings that are not sensitive to context. These models were trained on the same text corpus of approximately 1.2 billion tokens that were obtained from Wikipedia (https://dumps.wikimedia.org/zhwiki/latest/zhwiki-latest-pages-articles.xml.bz2.) with the same model parameters (i.e., window width of 2, negative number as 15 in fastText) (see Fig. 2c).
- **Contextual word embeddings** We adopted the BERT, ERNIE, GPT2 and Electra models as the contextual word embeddings that are sensitive to word context. Specifically, we extracted the 768-dimensional embeddings from all 12 layers of BERT (https://huggingface.co/bert-base-chinese), ERNIE (https://github.com/thunlp/ERNIE) and GPT2 (this was trained utilizing the corpus from https://github.com/CLUEbenchmark/CLUE and the model from https://github.com/Morizeyao/GPT2-Chinese), and the 256-dimensional embeddings formed 12 layers of Electra (https://github.com/google-research/electra). Following the method in Chersoni *et al*.^13^, we first randomly sampled at most 1,000 sentences for each target word from the aforementioned Wikipedia corpus. Then, we fed sentences to these embedding models and extracted the vectors from the output layer. Finally, we averaged these vectors from sentences of a target word as a contextual embedding for this target word (see Fig. 2c).
- **Visual embeddings** We used two ConvNet-based models, ResNet (https://huggingface.co/facebook/detr-resnet-101) and DenseNet (https://pytorch.org/vision/main/models/generated/torchvision.models.densenet169.html), and two transformer-based models, Vision Transformer (Vit) (https://huggingface.co/google/vit-base-patch16-224-in21k) and Bidirectional Encoder representation model (Beit) (https://huggingface.co/microsoft/beit-base-patch16-224-pt22k-ft22k) to compute image embeddings. Specifically, with six different images of a word as inputs separately, we extract the vectors from the last network layer and average them to obtain the image embeddings (see Fig. 2c).
- **Category** The word category was annotated by linguists based on the Harbin Institute of Technology. We also provided the part-of-speech (POS) tag of each word from PKU Chinese Treebank (see Fig. 2d).

In addition to the neuroimaging datasets, we provide rich information on the concept stimuli, including semantic features, various word embeddings and word categories:

- **Semantic features** As shown in Fig. 2a, the semantic feature includes 14 domains, i.e., vision, somatic, audition, gustation, olfaction, motor, spatial, temporal, causal, social, cognition, emotion, drive, and attention. Each domain involves several attributes (1–15). Three examples are shown in Fig. 2b. Consistent with intuition, the concept ‘教堂 (church)’ as a noun and objects received relatively higher ratings on sensory and motor domains, while the concepts ‘逃跑 (flee)’ and ‘安全 (safe)’ as verb and adjective, respectively, received relatively higher ratings on abstract domains.

## Data Records

**Fig. 3:**
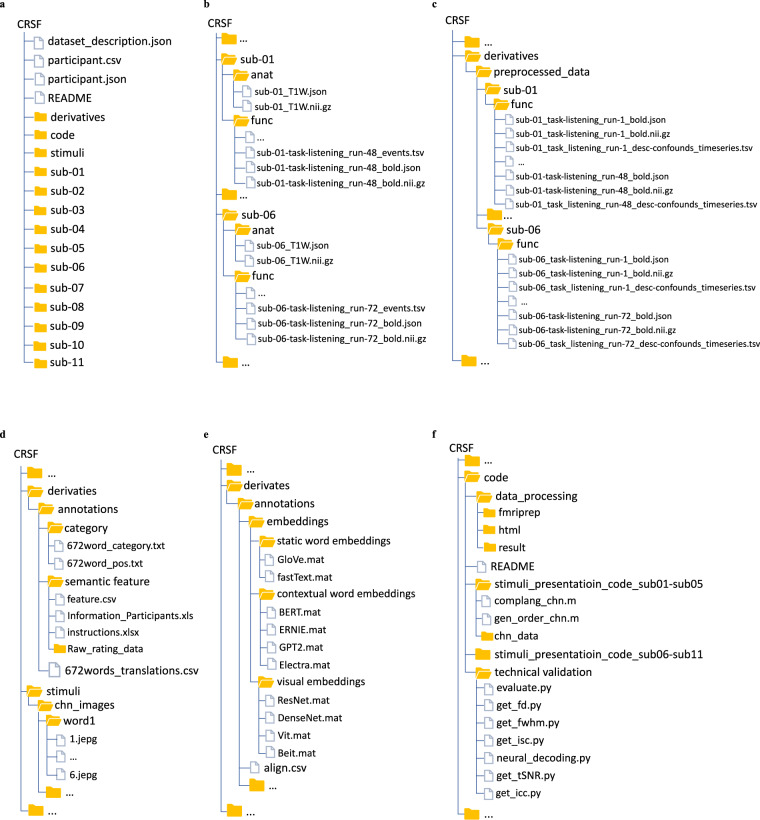
Organization of the data collection. (**a**) General overview of the directory structure. (**b**) Content of participant-specific raw data directories. (**c**) Content of participant-specific preprocessed data directories. (**d**) and (**e**) Content of the annotation directory, including category, semantic features and various word embeddings. (**f**) Codes used in this experiment.

The data collection using the BIDS data representation is available on the OpenNeuro platform at https://openneuro.org/datasets/ds004301^4^. As shown in Fig. 3a, our data include data description files, the raw fMRI data collected for each participant in the “sub-*“ folders, the code used in this experiment in the “code” folders, the preprocessed fMRI data plus various annotations in the “derivatives” folder, and the stimuli presentation of word image pair in the “stimuli” folder. More details about these folders are provided below.

### Participant folder

Each participant is stored in two subfolds, named “anat” and “func” (Fig. 3b). The “anat” folder includes the T1 MRI data, and the “func” folder includes functional MRI data in native space, consisting of 48 or 72 runs. The json files contain information about the acquisition parameters.

### Derivatives folder

The “derivatives/preprocessed_data” folder (Fig. 3c) contains the preprocessed fMRI data for each participant. The preprocessed fMRI data include all 48 or 72 runs in MNI spaces. The “derivatives/annotations” folder (Fig. 3d,e) contains word category, semantic features and various word embeddings.

### Stimuli folder

The stimuli words and pictures are provided in the “stimuli” folder (Fig. 3d). Note that some of the pictures have copyrights which are replaced with blank pictures. The original pictures are available upon request to the authors. In addition to this basic information, we provide rich annotations about the concept stimuli, including the word category, various word embeddings and semantic annotations.

### Code folder

The code for stimuli presentation during data collection, fMRI preprocessing and technical validation are provided in the “code” folder (Fig. 3f).

## Technical Validation

To validate the quality of the neuroimaging data, we checked head motions and inherent spatial smoothness and calculated the temporal signal-to-noise ratio (tSNR) and intersubject correlation (ISC) across all participants. To further verify that semantic information is encoded in the fMRI data, we conducted neural decoding experiments. Moreover, we checked the consistency of semantic ratings across participants by using intraclass correlation coefficients (ICC).

### Analysis of motion

The fMRIprep generates the frame-to-frame head displacement in three translational and three rotational directions after fMRI preprocessing. To evaluate the head motion of participants during each fMRI scan, we computed the FD by adding the absolute head displacement in all six directions. Moreover, since FD values greater than 0.2 mm are usually regarded as high head motion, we also calculated the percentage of frames with FD >0.2 mm in each run.

**Fig. 4:**
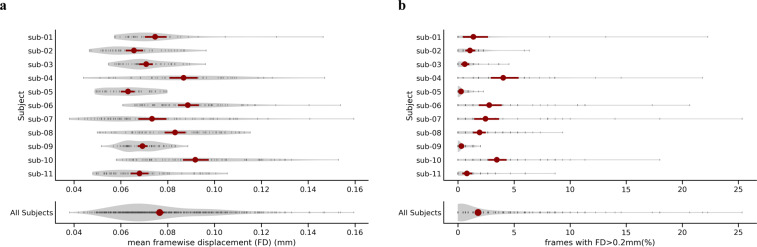
Results of FD for each participant. (**a**) Mean FD values for each run and each participant. (**b**) Percentage of frames where FD >0.2 mm for each run. The red marks are the mean FD and percentage across all runs, and the red bars represent the 95% bootstrap confidence interval.

As shown in Fig. 4a, the mean FD values for all participants are less than 0.2 mm, with minimum and maximum values of 0.04 mm and 0.16 mm, respectively. This suggests that all 11 participants had minimal head motion. Figure 4b shows that only four participants had slightly more than 20% of frames with FD > 0.2 mm in a few runs, which is comparable to or better than existing fMRI datasets^14,15^.

### Spatial smoothness

**Fig. 5:**
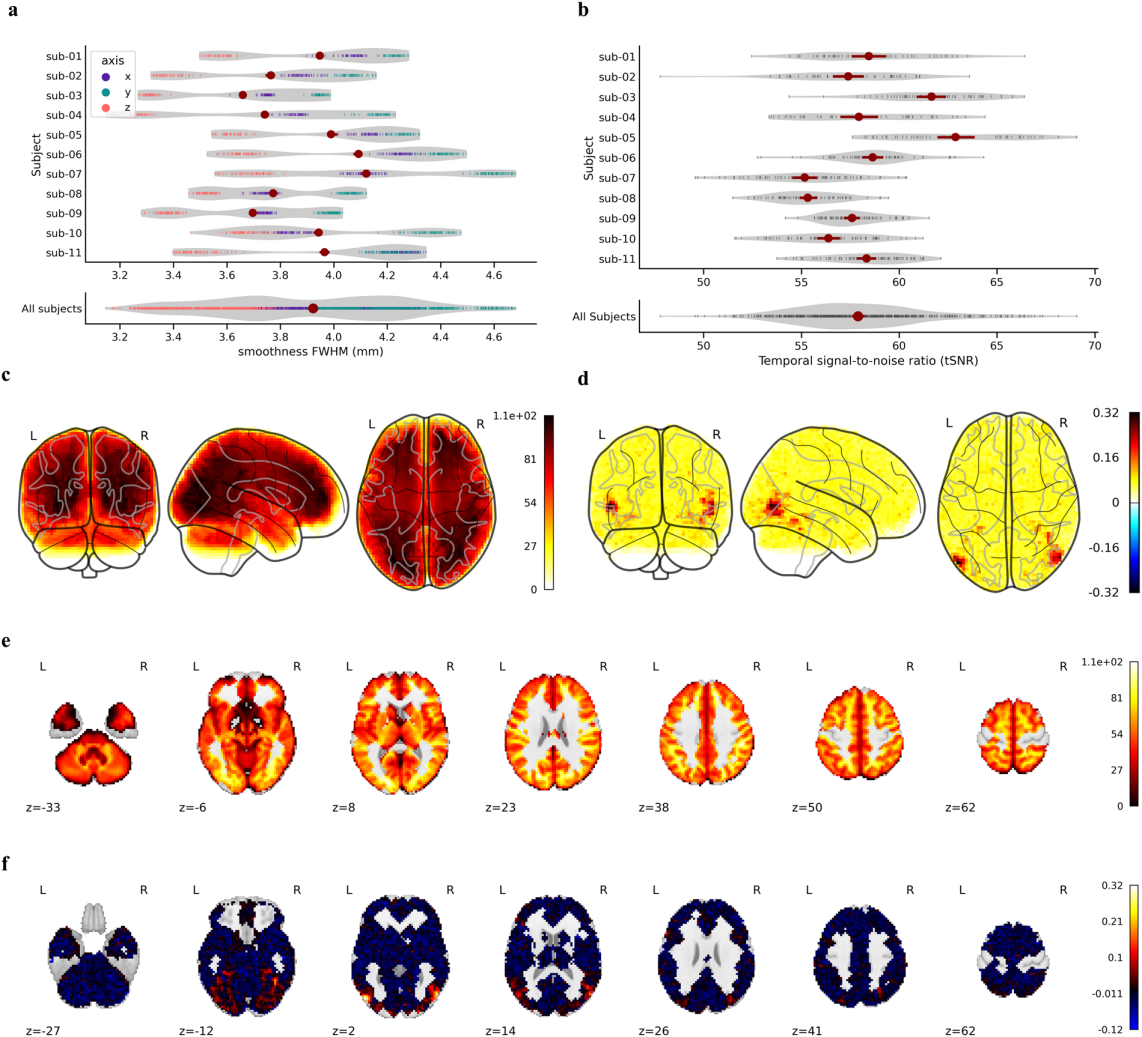
Results of fMRI technical validation. (**a**) Spatial smoothness in three directions for each run and each participant. (**b**) The mean tSNR for each run and participant. (**c**) Group-level mean tSNR in MNI space (glass brain). (**d**) Group-level mean ISC in the MNI space (glass brain). (**e**) Group-level mean tSNR in MNI space (axial direction). (**f**) Group-level mean ISC in the MNI space (axial direction).

The inherent spatial smoothness of the preprocessed fMRI data was quantified using the 3dFWHMx function in AFNI, which estimates the smoothness of the fMRI data in three directions (x-axis, i.e. left–right direction; y-axis, i.e. anterior–posterior direction; z-axis, i.e. inferior–superior direction). Specifically, the smoothness in each direction was computed as the ratio of the variance in that direction to the total variance across the image^16^ using the fMRI data without normalization. The BOLD time series temporarily detrended before smoothness estimation. As shown in Fig. 5a, the spatial smoothness within the same axes is similar across participants owing to the similar acquisition parameters applied with the same scanner models.

### tSNR of fMRI

To quantify the signal strength of fMRI, we calculated the temporal tSNR for each participant. Specifically, for each voxel, we used its temporal standard deviation to divide its temporal mean. The tSNR was computed using the preprocessed fMRI data for each run of each subject, and the group tSNR was generated by taking the mean tSNR value across all participants. As shown in Fig. 5b,c,e, the mean tSNR across all participants was 57.89 (SD = 3.18), which is comparable to or better than previous datasets. In addition, most brain regions have relatively high tSNR values.

### ISC of fMRI

ISC is often computed to evaluate the consistency of the brain responses to stimuli across multiple participants. To conduct the ISC analysis, we calculated the correlation between the brain response of one participant and the mean response of all the remaining participants. This procedure was conducted for all participants, yielding one ISC map for each participant. Then, we calculated an average map at the group level, which is shown in Fig. 5d,f. The brain regions located in the temporal lobe and the occipital lobe have high ISC values, which are conventionally related to language and vision processing.

### Neural decoding

Neural decoding learns a mapping from brain activation patterns to semantic concepts, that is, predicting word embeddings from brain activation^6,17^. To perform neural decoding, we first conducted a first-level analysis to generate a t-map for each word. Then, following Pereira *et al*.^18^, we selected the most informative 5,000 voxels and used the t value of these voxels as the brain activation features. Finally, we trained a ridge regression model to predict word embeddings from these brain activation features using cross-validation. The decoding results were assessed by pairwise classification accuracy, which was computed by comparing the similarity between the predicted word vectors and the original vectors of the right word and a random word.

**Fig. 6:**
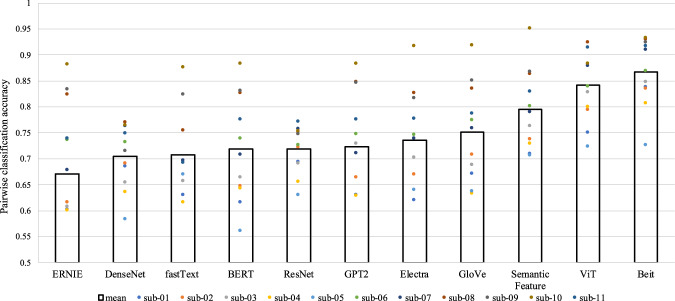
Pairwise classification results with different word embeddings.

In Fig. 6, we can see that all decoding models achieved the above-chance performance for all participants, indicating that the fMRI data encode semantic information.

### ICC of Semantic feature ratings

**Fig. 7:**
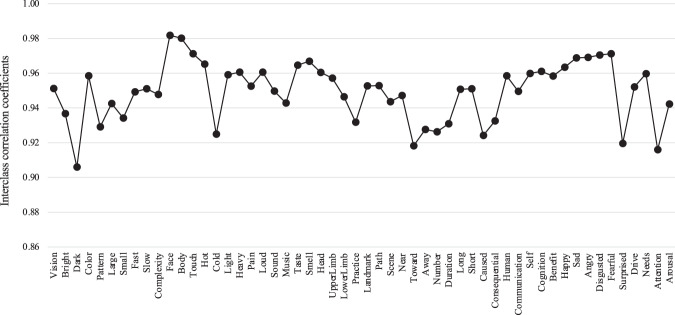
ICC results of all semantic features.

To assess the consistency of semantic ratings across participants, we calculated the ICC^19^, which compares the variability within a group to the variability across groups, that is, comparing the variability within semantic ratings of one participant to the variability across semantic ratings from all participants. The intuition is that if there are unreliable participants, the variability of those participants should be different than the variability of other reliable participants. To make our findings generalizable to other participants and words, we assumed that both participants and tests (672 words) are random factors and estimated the reliability with an average of k = 30 participants. Therefore, we selected the form of ICC(2,k) that uses a two-way random effect model with an average score^20^. The results are summarized in Fig. 7. For all semantic features, the ICCs were above 0.9, indicating good reliability of our ratings.

## Data Availability

The stimuli presentation, data preprocessing, and technical validation scripts are available at https://openneuro.org/datasets/ds004301^4^.
