# Author Correction: An fMRI Dataset for Concept Representation with Semantic Feature Annotations

**DOI:** 10.1038/s41597-023-02480-w

**Published:** 2023-08-23

**Authors:** Shaonan Wang, Yunhao Zhang, Xiaohan Zhang, Jingyuan Sun, Nan Lin, Jiajun Zhang, Chengqing Zong

**Affiliations:** 1 National Laboratory of Pattern Recognition, Institute of Automation, CAS, Beijing, China; 2 School of Artificial Intelligence, University of Chinese Academy of Sciences, Beijing, China; 3 CAS Key Laboratory of Behavioural Sciences, Institute of Psychology, Beijing, China; 4 Department of Psychology, University of Chinese Academy of Sciences, Beijing, China

**Keywords:** Neural encoding, Language, Neural encoding, Language

Correction to: *Scientific Data* 10.1038/s41597-022-01840-2, published online 24 November 2022.

The Acknowledgements section was missing from this article and should have read ‘This work is supported by Youth Innovation Promotion Association CAS.’

